# Influence of mesenchymal stem cells on stomach tissue engineering using small intestinal submucosa

**DOI:** 10.1002/term.1794

**Published:** 2013-08-04

**Authors:** Hiroki Nakatsu, Tomio Ueno, Atsunori Oga, Mitsuhiro Nakao, Taku Nishimura, Sei Kobayashi, Masaaki Oka

**Affiliations:** 1 Department of Digestive Surgery and Surgical Oncology (Department of Surgery II), Yamaguchi University Graduate School of MedicineJapan; 2 Department of Molecular Pathology, Yamaguchi University Graduate School of MedicineJapan; 3 Department of Molecular Physiology and Medical Bioregulation, Yamaguchi University Graduate School of MedicineJapan

**Keywords:** small intestinal submucosa, SIS, stomach tissue engineering, mesenchymal stem cell, extracellular matrix, GFP

## Abstract

Small intestinal submucosa (SIS) is a biodegradable collagen-rich matrix containing functional growth factors. We have previously reported encouraging outcomes for regeneration of an artificial defect in the rodent stomach using SIS grafts, although the muscular layer was diminutive. In this study, we investigated the feasibility of SIS in conjunction with mesenchymal stem cells (MSCs) for regeneration of the gastrointestinal tract. MSCs from the bone marrow of green fluorescence protein (GFP)-transgenic Sprague–Dawley (SD) rats were isolated and expanded *ex vivo*. A 1 cm whole-layer stomach defect in SD rats was repaired using: a plain SIS graft without MSCs (group 1, control); a plain SIS graft followed by intravenous injection of MSCs (group 2); a SIS graft co-cultured with MSCs (group 3); or a SIS sandwich containing an MSC sheet (group 4). Pharmacological, electrophysiological and immunohistochemical examination was performed to evaluate the regenerated stomach tissue. Contractility in response to a muscarinic receptor agonist, a nitric oxide precursor or electrical field stimulation was observed in all groups. SIS grafts seeded with MSCs (groups 3 and 4) appeared to support improved regeneration compared with SIS grafts not seeded with MSCs (groups 1 and 2), by enabling the development of well-structured smooth muscle layers of significantly increased length. GFP expression was detected in the regenerated interstitial tissue, with fibroblast-like cells in the seeded-SIS groups. SIS potently induced pharmacological and electrophysiological regeneration of the digestive tract, and seeded MSCs provided an enriched environment that supported tissue regeneration by the SIS graft in the engineered stomach. © 2013 The Authors. *Journal of Tissue Engineering and Regenerative Medicine* published by John Wiley & Sons, Ltd.

## 1. Introduction

Tissue-engineering and cell-therapy approaches are increasingly being developed for the treatment of tissue defects and injuries. Porcine-derived small intestinal submucosa (SIS) is an example of the types of bioscaffold that are currently being used for the regeneration of various gastrointestinal tissues in animals (Chen and Badylak, 2001; de la Fuente *et al*., 2003; Doede *et al*., 2009; Hoeppner *et al*., 2009; Lee *et al*., 2008; Nishimura *et al*., 2010; Qin and Dunn, 2011; Ueno *et al*., 2007a, 2007b; Wang *et al*., 2003, 2005). SIS is useful for such approaches because it is an acellular biodegradable collagen-rich matrix that contains ultrastructural features and functional growth factors similar to those in native submucosa (Hodde *et al*., 2007; McDevitt *et al*., 2003; Voytik-Harbin *et al*., 1997).

We previously demonstrated that commercial SIS grafts supported the regeneration and functional recovery of artificially-induced stomach and caecum wall defects in rats (Nishimura *et al*., 2010; Ueno *et al*., 2007a, 2007b). We observed sufficient regrowth of smooth muscle cells in the grafted tissue in the caecum but suboptimal regrowth in the stomach. To facilitate the effective regrowth of the smooth muscle layers, the seeding of cells on biodegradable scaffolds has demonstrated a beneficial impact on the remodelling of the bladder (Chung *et al*., 2005; Frimberger *et al*., 2005; Lu *et al*., 2005; Shukla *et al*., 2008; Wu *et al*., 2011; Zhang *et al*., 2004, 2005) and gastrointestinal tract (Araki *et al*., 2009; Hori *et al*., 2002; Nakase *et al*., 2006; Qin and Dunn, 2011). Recently, several studies have demonstrated the successful regeneration of bladder defects in animals using SIS grafts seeded with cells such as human embryonic germ cell-derived cells (Frimberger *et al*., 2005), muscle-derived cells (Lu *et al*., 2005; Zhang *et al*., 2004), urine-derived stem cells (Wu *et al*., 2011) or bone marrow stromal cells (Chung *et al*., 2005; Shukla *et al*., 2008; Zhang *et al*., 2005).

Because other researchers have successfully used SIS grafts seeded with various types of stem/progenitor cells to regenerate bladder defects, in the present study we developed a novel stomach tissue-engineering approach using commercialized SIS seeded with bone marrow-derived mesenchymal stem cells (MSCs) as the cell source. We used MSCs because they are autologous, relatively easy to harvest and exhibit multipotentiality, and are therefore of potential clinical use. It is reasonable to expect that a seeded SIS graft may promote the regeneration of smooth muscle cells in the gastrointestinal tract, because SIS is originally from the small intestine and may provide a niche containing the appropriate functional growth factors that support the differentiation of progenitor cells, such as MSCs, into native gastrointestinal components.

The objective of this study was to investigate the utility of SIS grafts in conjunction with MSCs from the bone marrow of transgenic rats expressing green fluorescent protein (GFP) for the regeneration of a whole-layer defect of the stomach. We also surveyed the fate of MSCs in engineered stomach tissue by immunohistochemistry for GFP.

## 2. Materials and methods

### 2.1. Laboratory animals

Forty Sprague–Dawley female rats, weighing 200–220 g (Japan SLC, Shizuoka, Japan), were used as the recipients of commercialized SIS grafts, with or without mesenchymal stem cells (MSCs), for the repair of a defect according to the following protocol. Ten 7 week-old GFP-transduced Sprague–Dawley male rats were used as allogeneic donors of bone marrow-derived MSCs. All rats were housed with free access to water and food under standard conditions (23 °C room temperature, 12 h dark–light cycles). Each animal was restricted with regard to food but not water 18 h prior to surgery. Experimental procedures were reviewed by the Committee of Ethics on Animal Experiments at Yamaguchi University School of Medicine, and performed in accordance with the Guideline for Animal Experiments in Yamaguchi University School of Medicine and the Law (No. 105) and Notification (No. 6) from the Japanese Government.

### 2.2. Isolation and culture of MSCs

The isolation of MSCs was performed according to a previously described method (Pittenger *et al*., 1999). In brief, GFP-transduced Sprague–Dawley rats were anaesthetized by intraperitoneal injection of 50 mg/kg pentobarbital (Kyoritsuseiyaku, Tokyo, Japan), and the bone marrow cells in the tibiae and femurs were aseptically flushed out with 10 ml normal culture medium. The normal culture medium consisted of Dulbecco's modified Eagle's medium (DMEM; Nissui Pharmaceutical Co., Tokyo, Japan) containing 10% fetal bovine serum (FBS; Gibco, New York, USA) and 1% penicillin–streptomycin (Gibco). After flushing, 20 ml normal culture medium was added to the solution of MSCs and the cells were divided into two 75 cm^2^ culture flasks (BD Falcon™, BD Biosciences). Both flasks were kept in a humidified 5% CO_2_ incubator at 37 °C for 72 h. The culture medium was changed every 2–3 days to remove non-adherent cells. The MSCs were subcultured when they reached ca. 80–90% confluence by washing once with phosphate-buffered saline (D-PBS^–^, Nissui Pharmaceutical Co.) and incubating with 0.25% trypsin–EDTA (Gibco) for 3–4 min at room temperature. After thorough detachment, the cells were suspended in normal culture medium and centrifuged once at 1000 × *g* for 5 min at room temperature. The cell pellets were resuspended in normal culture medium and subcultured in three T75 culture flasks until they were 80–90% confluent. Subsequent passages were performed according to the same protocol. MSCs at passages 3–4 were used for all experiments.

### 2.3. Experimental design and surgical manipulation

All 40 female Sprague–Dawley rats underwent the surgical procedure. Anaesthesia was performed by intraperitoneal injection of pentobarbital (30 mg/kg). A circular, full-thickness layer defect measuring 1 cm in diameter was created within the antrum (columnar epithelium region), which led to a 60–70% functional defect in the anterior stomach wall. The defect was secured with one of the prepared SIS grafts described below, using a continuous 5-0 polydioxanone suture (PDS-II, Ethicon, Tokyo, Japan), as described previously (Nishimura *et al*., 2010; Ueno *et al*., 2007a). For future reference, 8–10 interrupted 5-0 polypropylene sutures (Prolene, Ethicon) were created to delineate the grafted area. After the 6 months of healing inside the host rats, the animals were sacrificed and the entire stomach was excised. Two tissue preparations were then taken from within the reorganized area delineated by the polypropylene sutures. One specimen was used to examine the *in vitro* motility of the regenerated tissue, and the other was used for immunohistological analysis. Mechanical testing was performed only on that tissue-engineered segment.

### 2.4. Preparation of grafted SIS

Rats were randomly assigned to four experimental groups. Each stomach defect was patched with one of the following pretreated SIS grafts: plain SIS graft (group 1, control); plain SIS graft followed by intravenous injection of MSCs (group 2); SIS graft co-cultured with MSCs (group 3); or SIS sandwich containing an MSC sheet (group 4).

#### 2.4.1. Group 1: plain SIS graft without MSCs (control)

Commercially-prepared 1 × 2 cm four-ply SIS (Biodesigns™ Surgisis®, Cook Biotech, West LaFayette, IN, USA) was folded in the middle to make a 1 cm square. The four corners were cut off to make a 1 cm disc consisting of two pieces of Surgisis® (eight-ply SIS in total) that were connected tangentially. The prepared SIS was submerged in sterile saline for 15 min prior to implantation. A previously created 1 cm circular, full-thickness layer defect of the stomach was secured with the prepared SIS. Stitches were taken from the seromuscular layer and placed within 1 mm of the edge of the SIS graft with a continuous 5-0 polydioxanone suture.

#### 2.4.2. Group 2: SIS graft followed by intravenous injection of MSCs

MSCs at a density of 7–9 × 10 ^6^ cells in 1 ml D-PBS^–^ were prepared and slowly injected into the vena cava immediately after the closure of the defect with a plain SIS graft, in the same manner as described above. Before administration of the MSCs, the solution of MSCs was filtered through a nylon mesh with a 100 µm pore size (Cell Strainer, BD Biosciences) to prevent acute thrombosis. A cell viability analyser (Vi-cell XR, Beckman Coulter, CA, USA) was used to confirm that the number of cells and the cell diameter were stable before and after filtration.

#### 2.4.3. Group 3: SIS graft co-cultured with MSCs

MSCs at a density of 2 × 10^6^ cells/cm^3^ were prepared in normal culture medium. A 1 cm disk-shaped SIS consisting of two pieces of Surgisis, as described in group 1, was unfolded and placed into each well of a six-well plate (BD Falcon, NJ, USA). The SIS was moisturized with a small amount of normal culture medium just sufficient to cover the surface of the SIS graft to improve cell attachment. Culture medium containing MSCs was dropped carefully onto the inner surface of the SIS graft in each well to permit adequate seeding. The plate was kept in a humidified 5% CO_2_ incubator at 37 °C for 7 days. Spreading of the MSCs on the surface of the SIS graft was confirmed by phase-contrast microscopy (Olympus IX71, Olympus, Tokyo, Japan) before implantation. The SIS graft was refolded in the middle to make a 1 cm disk containing the co-cultured MSCs on the inside. The stomach defect was treated using SIS co-cultured with MSCs in the same manner as described above.

#### 2.4.4. Group 4: SIS sandwich containing an MSC sheet

MSCs at a density of 2 × 10^6^ cells/cm^3^ in normal culture medium were cultured on a 35 mm diameter temperature-responsive culture dish (Upcell®, CellSeed, Tokyo, Japan) at 37 °C for 4 days until confluent. The dish temperature was reduced from 37 °C to 20 °C for 1 h to enable the MSCs to spontaneously detach as a monolayered MSC sheet floating in the culture medium. The MSC sheet was gently sandwiched between plain SIS disks as described for group 1. The stomach defect was treated using the SIS sandwich containing an MSC sheet in the same manner as described above.

### 2.5. Muscle contractility *in vitro*

Dose- or frequency-response studies were conducted to assess the tissue response to drug dosage or stimulus potency in isolated tissue preparations. Tissue-organ baths filled with buffer were used for *in vitro* experiments to investigate the pharmacology and electrophysiology of the tissue specimens. The lengths and widths of the isolated tissue preparations were integrated before analysis. Both ends of the tissue preparations were suspended between two platinum electrodes in 10 ml tissue–organ baths (AD Instruments, Castle Hill, Australia). Krebs–Henseleit buffer (118 m m NaCl, 4.8 m m KCl, 2.5 m m CaCl_2_, 25 m m NaHCO_3_, 1.2 m m KH_2_PO_4_, 1.2 m m MgSO_4_ and 11 m m glucose) was used to maintain the integrity of the tissues for several hours in a temperature-controlled environment at 36 °C while physiological measurements were performed. Continuous bubbling with 95% O_2_ and 5% CO_2_ was performed throughout the experiment. Tissue preparations were adjusted at a resting load of 0.5 g and equilibrated for 1 h. Changes in mechanical contractility *in vitro* were recorded on a polygraph through isometric transducers (Nihon Kohden, Tokyo, Japan) and analysed using a computer-assisted system (Power Lab, AD Instruments, Castle Hill, Australia). A dose–response curve was obtained for each tissue preparation with carbachol hydrochloride (CCH, muscarinic receptor agonist; 1 × 10^–8^–10^–4^ m) and sodium nitroprusside (SNP, precursor of nitric oxide; 1 × 10^–7^–10^–4^ m). Activation of intrinsic nerves was achieved by electrical field stimulation (EFS; 50 mV, 1.0 ms duration, 10 s trains at 2.5, 5, 10, 20, 40 and 80 Hz) induced by an electric stimulator (Nihon Kohden). All chemicals were purchased from Wako Pure Chemical Industries (Osaka, Japan). Fresh stock solutions of CCH and SNP were routinely prepared in saline. Each stock solution was then further diluted to the appropriate concentration.

### 2.6. Histological evaluation

The second tissue specimens were fixed with 4% buffered paraformaldehyde (Wako Pure Chemical Industries), embedded in paraffin and sectioned at 4 µm along the circular muscle. Haematoxylin and eosin (H&E) staining was performed according to conventional methods. Immunohistochemistry was used to confirm the regeneration of smooth muscle, neural fibres and gastric parietal cells, as described previously (Nishimura *et al*., 2010), using antibodies against *α*-smooth muscle actin (anti-*α*-SMA, Abcam, Tokyo, Japan), desmin (Abcam), S-100 protein (MBL, Nagoya, Japan), and H^+^/K^+^-ATPase (Cosmo Bio, Tokyo, Japan). Anti-GFP antibody (MBL) was used to trace the MSCs by immunohistochemistry. The sections were incubated with primary antibodies (anti-*α*-SMA, 1:400; anti-desmin, 1:100; anti-S-100 protein, 1:1000; anti-H^+^/K^+^-ATPase, 1:100; anti-GFP, 1:400) in a humidified chamber at 37 °C for 40 min. After washing three times in wash buffer (Dako North America, CA, USA), the samples were incubated with secondary antibodies labelled with a polymer high-resolution pack (EnVision^+^, Dako North America) at 37 °C for 40 min. After washing three times they were developed with a DAB kit (EnVision^+^, Dako Japan, Kyoto, Japan). Finally, the samples were co-stained with haematoxylin for 5 min to visualize the cell nuclei. The thickness of the whole layer, the mucosa and the smooth muscle layer in each sample was measured in triplicate.

### 2.7. Data and statistical analysis

Data are expressed as mean ± standard error of the mean (SE). Statistical analyses were performed using two-way repeated analysis of variance (ANOVA) followed by the Bonferoni test; *p* < 0.05 was considered statistically significant.

## 3. Results

### 3.1. Clinical signs and macroscopic findings

All rats survived and thrived and were healthy until they were euthanized. On gross inspection, the SIS graft had disappeared and the area where the SIS graft was patched could only be established by identification of the remaining polypropylene sutures. No evidence of diverticula formation and/or shrinkage was apparent in the grafted region.

### 3.2. Muscle contractility *in vitro*

**Figure 1:**
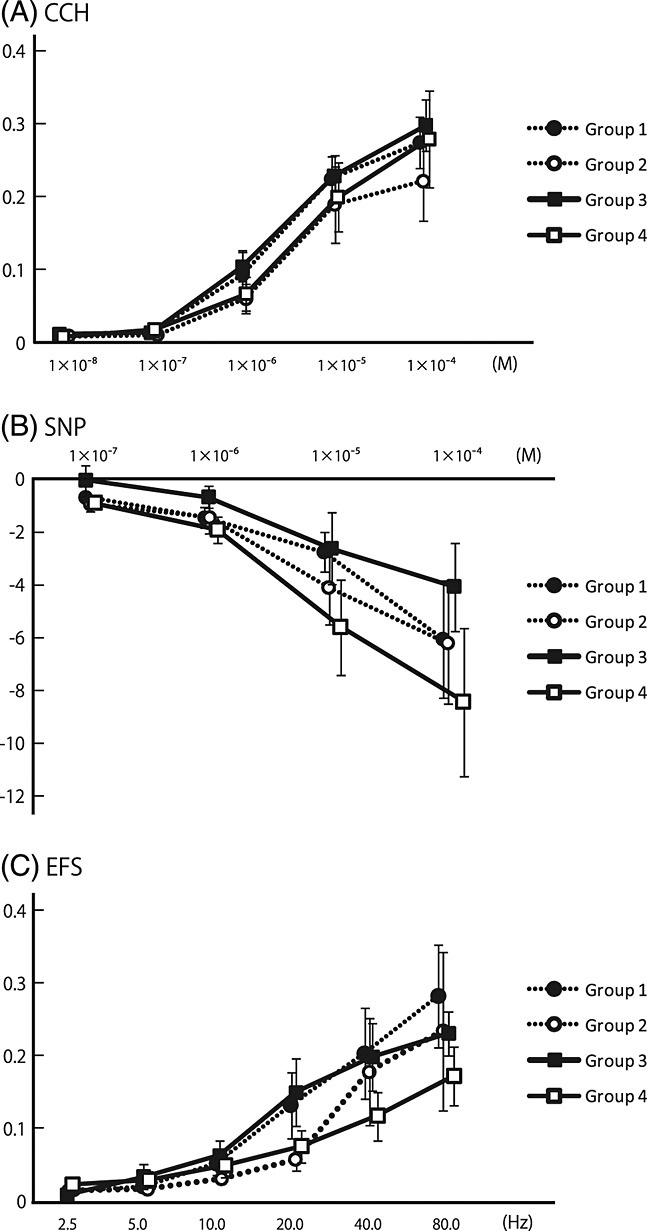
Muscle contractility *in vitro* in response to various stimuli at 6 months post-injury and implantation of the SIS graft. Each stimulus produced contraction or relaxation in a contraction- or frequency-dependent manner. Mean *in vitro* muscle contractility in response to a muscarinic receptor agonist, CCH (A), a nitric oxide precursor, SNP (B) or electrical field stimulation (EFS) (C) showed no significant differences among groups. Groups 1 (control) and 2 are not seeded with MSCs; groups 3 and 4 are seeded with MSCs

The outcomes of mean *in vitro* muscle contractility in response to various stimuli are shown in Figure1. CCH produced contraction in each group in a concentration-dependent manner. There was no significant difference in the amplitude of the response to CCH among groups, even at the highest stimulus concentration of 1 × 10^–4^ m. SNP induced relaxation in each group in a concentration-dependent manner. No significant difference was found in the amplitude of the response to SNP among groups. Each strip exhibited contraction in response to electrical field stimulation (EFS) in a frequency-dependent manner. The amplitude was not significantly different among groups.

### 3.3. Microscopic findings

**Figure 2:**
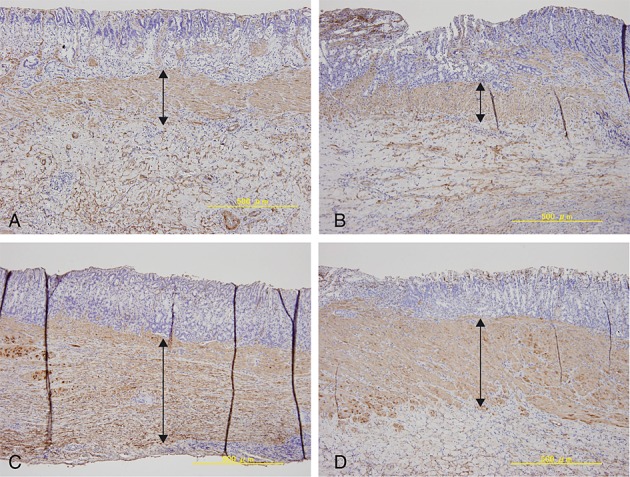
Immunohistochemical analysis of *α*-SMA expression in the regenerated stomach at 6 months post-injury and implantation of the SIS graft. Tissue defects secured with a plain SIS graft without MSCs (A; group 1, unseeded; control), with an SIS graft followed by intravenous injection of MSCs (B; group 2, unseeded), with an SIS graft co-cultured with MSCs (C; group 3, seeded) and with an SIS sandwich containing an MSC sheet (D; group 4, seeded). The bidirectional arrows indicate the thickness of the smooth muscle bundle

**Figure 3:**
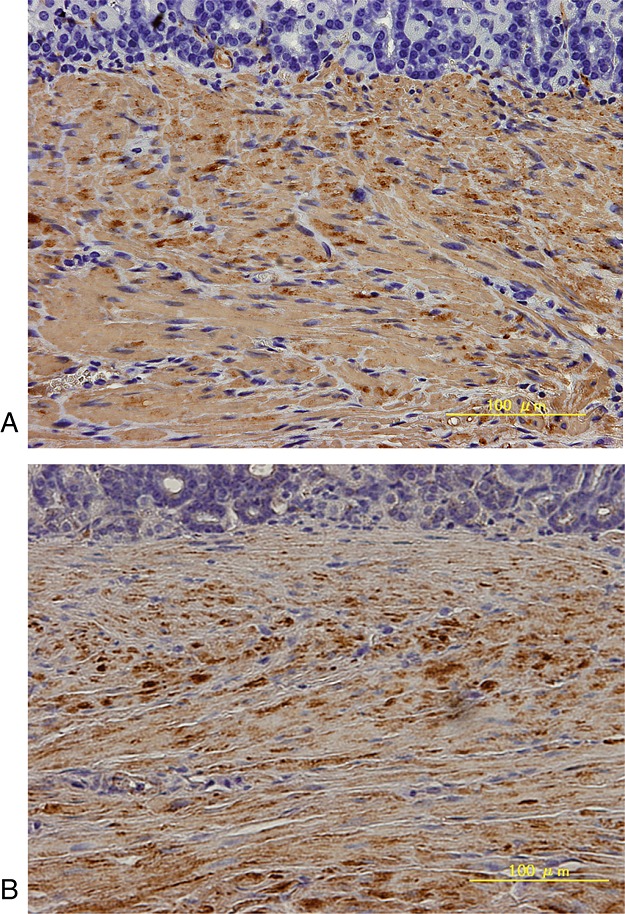
High-power micrograph showing immunohistochemical analysis of *α*-SMA and desmin expression in the seeded group at 6 months post-injury and implantation of the SIS graft. Staining with an anti-*α*-SMA antibody (A) and an anti-desmin (B) revealed the cells and structures of the submucosa and the muscularis propria. The directionality of the smooth muscle layer was aligned along the inner circular muscle layer

**Figure 4:**
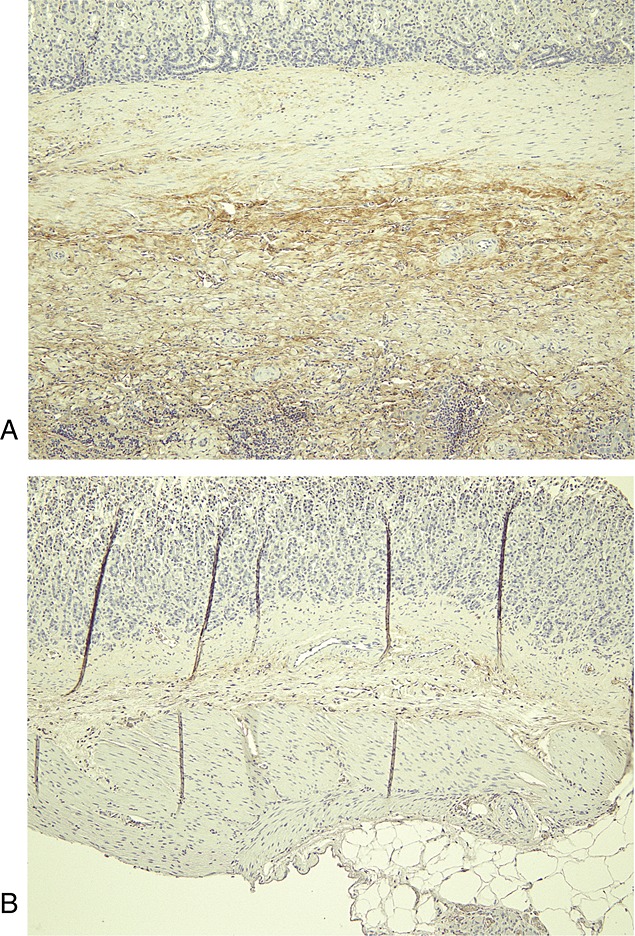
Immunohistochemical analysis of GFP staining at 6 months post-injury and implantation of the SIS graft. Staining with an anti-GFP antibody revealed the regenerated interstitial tissue but not the smooth muscle or mucosa of the regenerated stomach (A). The negative control was obtained using normal stomach tissue distal to the grafted area in the same section (B)

**Figure 5:**
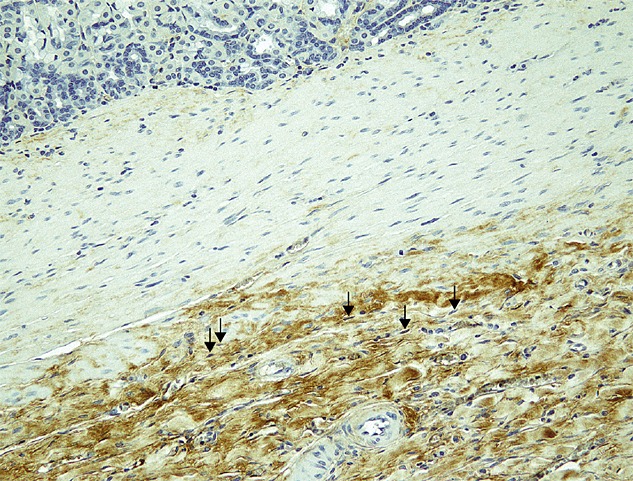
High-power micrograph showing immunohistochemical analysis of GFP expression in the seeded group at 6 months post-injury and implantation of the SIS graft. The regenerated tissue in groups 3 and 4 contained cells with spindle-shaped nuclei that resembled fibroblasts (arrowheads)

Repair of the whole-layer defect progressed favourably in all grafted animals. The inner lumen was re-epithelialized by poorly or moderately differentiated epithelial cells in 80% of the animals. Gastric parietal cells stained with anti-H^+^/K^+^-ATPase were also found in the re-epithelialized mucosa in most cases. The original SIS material had essentially disappeared, except for slight foreign-body reactions in some cases, and newly formed blood vessels were seen. The subserosal layer was thickened with fibrous tissue. In our model, inward bulging of the severed edges of the proper muscle layer was never observed, despite a full-thickness layer defect of the stomach wall, which is usually seen during the healing phase of deep peptic ulcers, due to scar contraction in the neighbouring area caused by disorganized arrangement of the fibrous scarring tissue. Repaired smooth muscle bundles were found in the inner portion of the SIS-regenerated wall, extending from muscularis mucosa or muscularis propria layer on the edge of the artificial defect. In groups 1 and 2, in which MSCs were not seeded, the grafted area exhibited thin smooth muscle bundle formation (Figure2A, B). By contrast, in groups 3 and 4, in which MSCs were seeded, well-structured smooth muscle layers were frequently observed (Figure2C, D), which appeared to develop continuously from the smooth muscle bundle in the native area. The directionality of the smooth muscle layer was aligned with the inner circular muscle layer, particularly in groups 3 and 4. Immunohistochemistry for both *α*-smooth muscle actin (*α*-SMA) and desmin highlighted the structures of the submucosa and the muscularis propria. A representative high-power view of the immunohistochemical appearance of anti-*α*-SMA and anti-desmin antigen in the seeded group is shown in Figure3. Immunostaining for S-100 protein highlighted structures scattered throughout the smooth muscle in the muscularis propria, as shown in our previous study (Ueno *et al*., 2007a). Anti-GFP antibody staining was apparent in the regenerated interstitial tissue in groups 3 and 4 (Figure4A), but not in the regenerated smooth muscle, neural tissue or mucosa. A negative control was obtained from normal stomach wall distal to the grafted area in the same specimen (Figure4B). GFP staining was also absent in groups 1 and 2 throughout both the regenerated tissue and the normal stomach tissue. The GFP-positive interstitial tissue in groups 3 and 4 contained cells with spindle-shaped nuclei that resembled fibroblasts (Figure5).

### 3.4. Dimensions of the regenerated smooth muscle bundles

**Figure 6:**
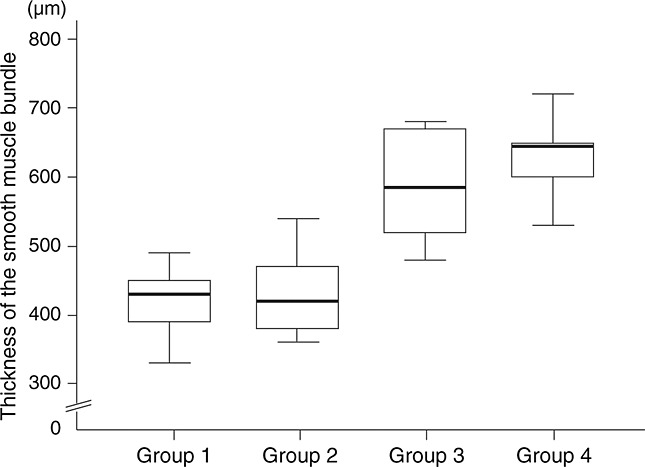
Dimensions of the regenerated smooth muscle bundle. Significant differences in the mean lengths of the smooth muscle bundles were observed between the seeded SIS groups (3 and 4) and the unseeded SIS groups (1 and 2)

The mean lengths of the smooth muscle bundles were 419.0 ± 14.3, 429.0 ± 18.8, 588.0 ± 24.3 and 638.0 ± 62.7 µm in groups 1, 2, 3 and 4, respectively. Significant differences in the mean length of the smooth muscle bundle were observed between the seeded SIS groups (3 and 4) and the unseeded SIS groups (1 and 2; Figure6). The mean lengths of the mucosa were 390.0 ± 22.1, 392.0 ± 24.9, 375.0 ± 23.8 and 398.0 ± 16.0 µm in groups 1, 2, 3 and 4, respectively. No significant difference was observed among groups. The mean lengths of the whole layers were 1574.0 ± 67.5, 1565.0 ± 73.2, 1531.0 ± 89.1 and 1585.0 ± 49.3 µm in groups 1, 2, 3 and 4, respectively. No significant difference was observed among groups.

## 4. Discussion

The ideal scaffold supports cell growth and acts as a matrix, allowing three-dimensional (3D) regrowth and the homing of different cell populations. The SIS graft is considered to be a suitable scaffold for gastrointestinal restoration because it is an acellular biodegradable collagen-rich matrix containing functional growth factors, such as basic fibroblast growth factor (bFGF), vascular endothelial growth factor (VEGF) and transforming growth factor-*β* (TGF*β*), which are considered vital to the regenerative process (Hodde *et al*., 2007; McDevitt *et al*., 2003; Voytik-Harbin *et al*., 1997). SIS has been commonly used as a bioscaffold for the replacement of various gastrointestinal tracts in animals, including the oesophagus (Doede *et al*., 2009), stomach (de la Fuente *et al*., 2003; Nishimura *et al*., 2010; Ueno *et al*., 2007a), small intestine (Chen and Badylak, 2001; Lee *et al*., 2008; Qin and Dunn, 2011; Wang *et al*., 2003) and colon (Hoeppner *et al*., 2009; Ueno *et al*., 2007b). We previously published the first report to show that plain SIS grafts induced the regeneration of an artificial defect in the rodent stomach with *in vivo* and *in vitro* functional recovery (Nishimura *et al*., 2010; Ueno *et al*., 2007a). However, the regrowth of the smooth muscle cells, which were located mainly in the submucosal layer, was inadequate, indicating that further improvements of the technique were required.

To accomplish the effective regrowth of the smooth muscle layers, the current technique of seeding cells on biodegradable scaffolds has been popularized for remodelling the bladder (Chung *et al*., 2005; Frimberger *et al*., 2005; Lu *et al*., 2005; Shukla *et al*., 2008; Wu *et al*., 2011; Zhang *et al*., 2004, 2005) or gastrointestinal tract (Araki *et al*., 2009; Hori *et al*., 2002; Nakase *et al*., 2006; Qin and Dunn, 2011). Recently, several studies have demonstrated successful smooth muscle regeneration by bladder tissue engineering in animals using SIS grafts seeded with cells, including embryonic stem cells (Frimberger *et al*., 2005), bone marrow stem cells (Chung *et al*., 2005; Shukla *et al*., 2008; Zhang *et al*., 2005) and tissue stem cells (Lu *et al*., 2005; Wu *et al*., 2011; Zhang *et al*., 2004). In particular, Frimberger *et al*. (2005) reported almost perfect regeneration of the rodent bladder, including epithelium, submucosa and muscle, using SIS seeded with human embryonic germ cells. Mesenchymal stem cells (MSCs) are an attractive alternative cell source to embryonic stem cells because they are obtained from the individual receiving the graft, and because cell procurement and expansion *ex vivo* are practicable. Kanematsu *et al*. (2005) speculated that some bone-marrow MSCs differentiated into smooth muscle on acellular matrix grafts in response to an environment created by native smooth muscle cells in engineered bladder. Also, Shukla *et al*. (2008) reported that they could obtain smooth muscle-like cells *in vitro* from bone-marrow MSCs. Furthermore, from the perspective of extracellular matrices, the SIS graft would be expected to advance the regeneration of smooth muscle cells if it was combined with MSCs for the regeneration of the gastrointestinal tract, because SIS is originally from the small intestine and may provide a niche with the appropriate ultrastructural features and functional growth factors to promote the differentiation of progenitor cells into native gastrointestinal components. Therefore, we examined the utility of SIS in conjunction with MSCs to recapitulate the stomach wall both histologically and functionally.

In our protocol, we utilized MSCs in three different procedures. Because an intravenous injection of MSCs was used to promote gastric ulcer healing in rats (Hayashi *et al*., 2008; Komori *et al*., 2005), we applied this method to group 2. According to these reports, bone marrow-derived cells are involved in the regeneration of the stomach after the induction of ulcers with ethanol in rats. We examined whether the systemically delivered MSCs were trapped in the liver, spleen or lungs before starting our study. We could not detect any GFP fluorescence in these organs in group 2 (data not shown). In group 3, cell seeding was performed according to the procedure of Frimberger *et al*. (2005), who demonstrated promising regeneration of the bladder as described above, although the source of cells was quite different. The cell sheet-based tissue engineering technique used in group 4 provides another approach to assist 3D tissue remodelling (Okano *et al*., 1993), which has enabled success in corneal reconstruction clinically (Nishida *et al*., 2004) and MSC-sheet transplantation to improve cardiac diastolic function following myocardial infarction in a preclinical model (Miyahara *et al*., 2006). As reported previously (Miyahara *et al*., 2006), we were able to obtain an MSC sheet using a temperature-responsive culture dish to prevent the use of proteolytic enzymes. Using a sandwiched SIS graft containing an MSC sheet is our original idea.

In the current investigation, we applied a novel stomach tissue-engineering approach to repair a whole-layer defect in rats, using commercially available SIS in combination with allo-MSCs expanded *ex vivo* from the bone marrow of GFP-transgenic rats. We also traced GFP by immunohistochemistry to determine the fate of MSCs in the engineered stomach tissue. Our results demonstrated the functional recovery of the grafted area by contractility in response to a muscarinic receptor agonist, a nitric oxide precursor or electrical field stimulation, regardless of whether the SIS graft was seeded with MSCs or the MSCs were injected after the SIS was grafted. These results imply that SIS may potently induce pharmacological and electrophysiological regeneration of the digestive tract with high reproducibility, as in our previous series (Nishimura *et al*., 2010; Ueno *et al*., 2007•). We presumed that insufficient contractility was obtained in both the seeded and unseeded groups because of the fibrous tissue in the subserosal layer.

Microscopically, however, the SIS grafts seeded with MSCs prior to transplantation appeared to support improved regeneration compared with grafts not seeded with MSCs, by enabling the development of well-structured smooth muscle layers. The actual length of the smooth muscle layer was observed to be significantly greater in seeded SIS groups than in unseeded SIS groups, and immunohistochemical staining with anti-*α*-SMA and anti-desmin showed a definite difference in cell quantity and tissue quality between the seeded and unseeded SIS groups. Our failure to identify GFP-positive smooth muscle cells in the regenerated stomach implies that, contrary to expectations, the regenerated smooth muscle cells possibly did not originate from the seeded MSCs. This finding is consistent with previous studies on bladder tissue engineering using SIS with MSCs by Shukla *et al*. (2008), who reported that MSCs within SIS may assist tissue regeneration in augmentation cystoplasty but may not significantly incorporate into smooth muscle bundles.

Tracing the transplanted MSCs by heterologous GFP expression indicated the presence of GFP in the regenerated interstitial tissue with fibroblast-like cells in the seeded SIS groups only. We stained sections obtained from the same block used for anti-GFP antibody staining with an antibody against *α*-smooth muscle actin. The layers stained with anti-GFP antibody in Figures4A and 5 were not stained with the antibody against *α*-smooth muscle actin (data not shown). Therefore, we do not believe that the GFP-positive cells differentiated directly into the smooth muscle cells. However, GFP-positive interstitial tissue did spread in the remodelled tissue in the seeded SIS groups, indicating that MSCs facilitate the repair of injured tissue by neighbouring native cells, including fibroblasts. It is possible that cell–cell interactions might be involved in wound healing by SIS and MSCs, as shown by Shukla *et al*. (2008) and Kanematsu *et al*. (2005). Caplan and Dennis (2006) have shown that the secretion of a variety of cytokines and growth factors by MSCs suppress the local immune system, inhibit scar formation and apoptosis, enhance angiogenesis and stimulate mitosis and differentiation in tissue-intrinsic reparative or stem cells. It remains unclear, however, which parts of the SIS microenvironment regulate transplanted MSCs. Although incidental detection of the scavenging of dead MSCs by host macrophages contributed to the positive staining (Frimberger *et al*., 2005), our results suggest that GFP-positive cells appear to contribute to tissue remodelling through the production of reasonable amounts of interstitial tissue when combined with an ideal extracellular matrix component (SIS) in the engineered stomach. Because SIS is derived from porcine intestinal tissue, further studies using SIS seeded with autologous MSCs for the repair of defects in the small intestine in swine could verify the hypothesis.

In addition to its impact on tissue engineering of the gastrointestinal tract, we believe that our study provides another example of the value of combined tissue-engineering and cell-therapy approaches to regenerative medicine. This approach is of particular interest for applications in which autologous MSCs can be used for patient treatment, which may increase the success rate of tissue-engineered therapies.
